# Knowledge, risk perception and adherence to COVID-19 prevention advisory among police officers in Makurdi Metropolis Benue State, 2020

**DOI:** 10.11604/pamj.2021.38.199.25664

**Published:** 2021-02-22

**Authors:** Ubong Akpan Okon, Christiana Onche, Simeon Whenayon Ajisegiri, Uche Katchy, Peter Onyema, Charles Uwazie, Isika Anastasia, Araga Abdullahi, Muhammad Shakir Balogun

**Affiliations:** 1 Nigeria Field Epidemiology and Laboratory Training Program, Abuja, Nigeria,; 2 Department of Public Health, Nigeria Police Medical Service, Makurdi, Nigeria,; 3 The George Institute for Global Health, University of New South Wales, Sydney, Australia,; 4 Lobb Gott Global Resources, Alberta, Canada,; 5 Department of Community Medicine, University of Calabar, Calabar, Nigeria

**Keywords:** COVID-19, risk perception, knowledge, adherence

## Abstract

**Introduction:**

the current pandemic of coronavirus disease (COVID-19) caused by a novel strain (SARS-CoV-2) is enormous and continues to pose a threat to the lives of people. In Nigeria, as of 21^st^ April 2020, 668 confirmed cases, 22 deaths and 188 recoveries have been reported. Police officers are at the forefront of enforcing advisories to ensure public compliance. However, there is a paucity of data on knowledge, risk perception, and adherence to COVID-19 advisories issued by the Health authorities particularly among the police officers. We, therefore, assessed the knowledge, risk perceptions and adherence to NCDC recommended advisory on COVID-19.

**Methods:**

we conducted a two-stage sampling cross-sectional study among different cadres of police officers in Benue State, Nigeria using a pretested, semi-structured, interviewer-administered questionnaire. The results of the study were presented in frequencies and proportions. Chi-square test was used for an association between variables at p-value < 0.05.

**Results:**

the mean age of the 305 participants was 39.1 ± 8.4 years and most, 124 (40.7%) of the participants were within age-group 30-39 years, 19 (64.3%.8) were male, 250 (82.0%) were married and 160 (52.5%) had secondary education as the highest qualification. Majority of the participants, 301 (98.7%) have heard about COVID-19 and the commonest source of information was via television/radio, 230 (76.4%). Most participants demonstrate a good knowledge of COVID-19 infection, 302 (99.0%) and positive risk perception of COVID-19, 303 (99.3%) but few demonstrated good adherences on COVID-19 prevention practices, 133 (43.6%). Participants’ academic qualification (X^2^ = 10.98, p = 0.001) and cadre (X^2^ = 112.5, p = 0.001) were found to be associated with good adherence.

**Conclusion:**

while most participants had a good knowledge of COVID-19 transmission dynamics, and positive risk perception about COVID-19, good adherence to public health advisories were low. We recommended periodic training, provision of adequate PPE and personal hand-sanitizers as a strategy to improve adherence.

## Introduction

The novel coronavirus pandemic has been described by the World Bank as the worst pandemic in 100 years [1]. Thousands of lives have been lost within days, health systems have been crippled and world traffic and trade has been stalled [1, 2]. Globally, as of 30^th^ of May, 2020, the World Health Organization (WHO) reported more than 5.8 million confirmed cases of COVID-19 in over 210 countries, with case fatality of 6.23% [2]. Of this, the region of the Americas accounted for the highest number of cases, with over 2.6 million people affected and case fatality of 5.77% [2]. The European region recorded the second highest number of 2.1 million cases with the overall highest case fatality of 8.45% [2].

Across the African continent, the COVID -19 pandemic continues to evolve rapidly with over 52 countries affected [2]. Currently there is an over 500% increase of confirmed cases between April to May 2020. Over 22,579 confirmed cases and more than 1,128 deaths have been recorded. Africa 96 902 cases 2 482 deaths. In West Africa, after South Africa and Ghana, Nigeria reported the third highest cases with 9,302 confirmed cases and 261 deaths. Of this, Lagos in the south-west zone with 5,13 cases had the highest number, followed by Kano with 958 cases and Abuja in the North-central zone with 89,674 cases [3]. Benue State, also in the north-central, currently has 9 cases with no testing center [3, 4]. Benue State is critical because it is a relaxation hub and overnight spot for travellers to the far north and southern parts of the country. It is officially known as the food basket of the nation [5] has major farm markets like yams, oranges and goats that attracts people from all parts of the country. Although movement is restricted within and along borders, market and other essential services remain open. It is critical that the state has adequate preparedness and response plan because of the significant traffic and trade in the region.

COVID-19 is caused by Severe Acute Respiratory Syndrome Coronavirus 2 (SARS-CoV-2) [6, 7], a virus that is genetically related to the coronavirus responsible for the SARS outbreak in 2003 [6, 7]. Genomic and phylogenetic analyses of SARS-CoV-2 sequences suggest COVID-19 is a zoonotic disease, with bats being the original animal reservoir [6, 7]. Transmission is via direct contact through respiratory droplets within one meters of individuals when an infected person coughing, sneezing, or talking comes in contact with mucosal surfaces, such as the eyes, nose or mouth Also, indirect transmission via contaminated fomites have been recorded [8, 9]. The spectrum of illness for COVID-19 ranges from mild respiratory symptoms like fever, cough and difficulty breathing, mimicking common cold, pneumonia and other respiratory diseases to more severe forms including end organ damage and shock [9, 10]. It is worth noting that other atypical symptoms like anosmia and dysgeusia have been recorded [11].

The management of COVID-19 is largely supportive as the quest for a potent and safe vaccine as well as specific antiviral treatment is underway [12-15]. The troubling combination of lack of specific treatment, absence of vaccines and huge number of asymptomatic cases accentuate the need for stringent preventive measures [13, 15, 16]. The World Health Organization has urged all countries to intensify their level of preparedness, alert, and response strategy to promptly identify and manage new cases of COVID-19 [16]. In response to this call, Nigeria Center for Disease Prevention and Control issued public health advisories centered on disease recognition, hand hygiene and social distancing [8]. Proper cough and sneezing etiquettes and appropriate use and disposal of face masks [9]. Other efforts to prevent spread of the virus include travel restrictions, quarantines, curfews, workplace hazard control and restriction of public gatherings have been instituted [8].

However, even with the best public health control efforts, advisories are not always readily adhered to by the public [17]. Knowledge of disease aetiology, mode of transmission, and treatment options is more likely to promote positive health-seeking behaviour [18]. Diseases that are perceived as serious are associated with better adherence to public health advisories compared to others perceived as less serious. Reciprocal determinism, a key construct of the social cognitive theory states that a person can both be an agent for change and a responder to change [19]. This concept is well suited for police officers who are frontline workers in this pandemic control. Not only do police officers enforce compliance on restriction of movements, but they also ensure other public health advisories along borders and within cities are maintained. If the police are seen wearing their face mask and maintaining social distancing, the principles of observational learning in the social cognitive theory will be made evident [19] and with the good knowledge on the risk factors, preventive measures as well as appropriate risk perception and adherence behaviour, they are likely to serve as agents of change, educating and motivating people in the course of their duty. Thus, many more people are likely to adhere to control efforts and consequently, halt and begin to reverse the current pandemic trend [12, 20]. Therefore, this study was carried out to assess the knowledge, risk perception and adherence to COVID-19 prevention advisory among police officers in Makurdi metropolis, Benue State.

## Methods

**Study area:** the study was conducted in Benue State Command Metropolis in North-central Nigeria. Benue State shares boundaries with five other states namely Nasarawa State to the north, Taraba to the east, Cross River State to the south, Enugu State to the south-west and Kogi to the west. Benue State has recorded three COVID-19 cases and shares a common boundary with the Republic of Cameroon on the south east that has recorded over 1000 COVID-19 cases. Benue occupies a landmass of 34,059 square kilometers and has Makurdi as the Capital City and 23 Local Government Areas. Makurdi has a population of 407,257 according to 2020 projected population. Benue State Police command, Makurdi Metropolis consist of 6 divisional headquarters, one area Command, the zonal headquarters, Mobile force, SPU and the State Headquarter. The command consists of different department such as the Finance and Administration, Investigation, Operation, Training, Communication, Work, Logistic and Supply.

**Study design:** it was a cross-sectional study.

**Study population:** the study comprised police officers, men and women of different cadres ranging from the superintendent cadre, inspector cadre and the rank-and-file cadre working and living in any of the police formation within Makurdi metropolis and available at the time of team visit.

**Eligibility criteria:** police officers working at the selected formation or beat and available at the time of team visit.

**Sampling technique:** two-stage sampling technique was used in the study. All the division within the Makurdi metropolis, Operational and Investigational units were included in the study. Sample sizes were allocated in proportion to size and participants were recruited consecutively until the required sample size was reached.

**Sample size calculation:** the sample size was calculated using Epi Info software using an estimated population of police officers within the metropolis as 1800. The confidence level of 95%, error of margin of 5% and 1% design effect was used. Fifty percent (50%) was used as the proportion of police officers with good knowledge of COVID-19. The sample size of 317 participants was calculated however after compensating for non-response rate of 10%, the sample size came to 348.

**Data collection:** data was collected using a pretested, semi-structured, interviewer-administered electronic questionnaire using the Open Data Kit (ODK) enabled on Android phone. The data was collected by sixteen trained health workers who served as a research assistant in 5 days. The research assistants approached respondents in their offices, check points including those on guards and administered the questionnaire based on the eligibility criteria. There were four sections of which, Section A covered socio-demographic characteristics of respondents such as age, sex, marital status, educational status, religious status and family size. Section B dealt on the Respondent Knowledge on the COVID-19 such as the cause of COVID-19, mode of transmission and prevention of COVID-19. Section C covered the risk perception on the COVID-19 infection while Section D dealt on adherence to COVID-19 prevention advisories. The questionnaire was pretested among 25 officers working in the section that were not included in the study were interviewed. Data analysis was done to clarify and correct ambiguities.

**Data analysis:** knowledge of COVID-19 was assessed using 30 questions requiring a “yes or no” response. The questions covered knowledge of COVID-19, modes and sources of transmission, symptoms of COVID-19 and preventive measures. The correct response was allocated 1 point while wrong one had no point. The scores were combined to form a composite score having a maximum score of 30 points. This composite score was converted to a percentage. Score ≤ 50% was considered poor knowledge while above 50% were rated as having good knowledge [21]. Risk perception was assessed using 22 questions requiring a yes or no response. The correct response was allocated 1 point while wrong one had no point. The scores were combined to form a composite score having a maximum score of 22 points and a minimum of zero. This composite score was converted to a percentage. Score ≤ 50% was considered negative perception while those who scored above 50% were rated as having positive perception [21]. Adherence was assessed using 9 questions requiring a yes or no response.

The correct response was allocated 1 point while wrong one had no point. The scores were combined to form a composite score having a maximum score of 9 points and a minimum of zero. This composite score was converted to a percentage. Score ≤ 50% was considered poor Adherence while those above 50% were rated as having good adherence [21]. Data were analyzed using STATA SE 64 statistics analysis software. The results of the study were presented in frequencies and proportion. Chi-square test was used for association between these proportions, and the p-value was set at < 0.05. The outcome (dependent) variable was the graded as level of practices of the respondents. The Fisher´s exact test was read in some of the test results as appropriate.

**Ethical considerations:** the study was approved by Benue State Ministry of Health Human Research Ethical Review Board with the reference number MOH/OFF3/VOL4/P97, Permission to conduct the study was obtained from the Police authority in Benue State and Study participants gave their written electronic consent after receiving detailed information after the study. Participants had to answer a yes-no question to confirm their willingness to participate voluntarily.

## Results

The mean age of the 305 participants was 39.1 ± 8.4 years and 124 (40.7%) of the participants were within the age-group 30-39 years. Majority of the respondents were male 196 (64.3%), 250 (82.0%) were married, 160 (52.5%) had secondary education as highest qualification and103 (33.8% were on the inspectorate cadre (Table 1).

**Table 1:** demographic characteristics of the police officer in Benue State Command, 2020

Demographic characteristics	Frequency (percent)
**Age group (years)**	
≤19	1 (0.3)
20-29	40 (13.1)
30-39	124 (40.7)
40-49	95 (31.1)
50-59	45 (14.8)
**Sex**	
Male	196(64.3)
Female	109(35.7)
**Marital status**	
Married	250 (82.0)
Single/divorced/widow	49 (16.07)
Co-habiting	6 (1.97)
**Religious status**	
Christianity	281(92.1)
Islamic	22 (7.21)
Others	2 (0.7)
**Highest academic status**	
Primary	2 (0.66)
Secondary	160 (52.5)
Tertiary	143 (46.7)
**Family size**	
1-5	165 (54.1)
6-10	131 (42.9)
=>10	9 (3.0)
**Cadre**	
Commissioner	4 (1.3)
Superintendent	54 (17.7)
Inspectorate	103 (33.8)
Rank and file	144 (47.2)

Majority of the participants, 301 (98.7%) have heard about COVID-19 and the commonest source of information was via television/radio, 230 (76.4%). Most participants, 199 (65.3%) believed that any age-group can be affected by COVID-19, 200 (65.6) believed that COVID-19 is caused by viral infection, 90 (29.5%) mentioned that there´s cure for COVID-19 while 258 (84.6%) claimed they know where to report a suspected case of COVID-19 (Table 2).

**Table 2:** police officers in Benue State Commands knowledge on COVID-19, 2020

Knowledge Variables	Frequency (%) (N = 305)
**Ever heard of COVID-19**	
Yes	301 (98.7)
**Source of information about COVID-19 (N = 301)**	
TV/Radio	230 (76.4)
Newspaper	13 (4.3)
Internet	7 (2.33)
Social media	45 (15.0)
Healthcare workers/clinic	1 (0.3)
Friends/peers	4 (1.3)
Family Members	1 (0.3)
Public Lecture	0 (0.0)
**Age group mostly affected**	
Children	3 (1.0)
Adolescence	37 (12.1)
Adult	0 (0.0)
Elderly	66 (21.6)
All age group	199 (65.3)
**Causes of COVID-19 Infection**	
Viral infection	200 (65.6)
Bacterial infection	11 (3.6)
Punishment from God	8 (2.6)
Witches and wizard	3 (1.0)
Curse from God	83 (27.2)
I don’t know	0 (0.0)
**COVID-19 infection has a cure**	
Yes	90 (29.5)
No	117 (38.4)
I don’t know	98 (32.1)
**COVID-19 infected person may not show symptoms**	
Yes	229 (75.1)
No	76 (24.9)
**Knowledge of where to report suspected case of COVID-19**	
Yes	258 (84.6)
No	47 (15.4)
**Have official contact detail of where to report suspected case of COVID-19**	
Yes	216 (83.1)
No	44 (16.9)

Most participants mentioned cough, 301 (98.7%), difficulty in breathing, 300 (98.4%) and fever, 283 (92.8%) as the symptoms of COVID-19 while droplet infections, 261 (85.6%), shaking of an infected person, 298 (97.7%) and contact of infected hands with the face, 289 (94.8%) were commonly mentioned as the mode of spread of COVID-19 (Table 3). Among the participants, 292 (95.7%) believed that they could be infected with COVID-19 while 282 (93.1%) believed COVID-19 is preventable. Avoiding crowded areas, 298 (97.7%), avoiding touching one´s eye, nose and mouth, 295 (96.7%) and avoiding contact with contaminated surfaces, objects or items of personal use, 294 (96.4%) were the most common prevention method perceived by the participants. Most participants, 299 (98.0%) were also willing to change their ways of life to prevent being infected with COVID-19 (Table 4). Majority of the participants, 284 (93.1%) said they observed regularly hand wash with soap and water, of which 245 (86.3%) said that wash their hand more than twice a day. Few participants own a pair of hand gloves, 83 (27.2%), face mask, 107 (35.1%) and hand sanitizer, 175 (57.4%) (Table 5).

**Table 3:** respondents’ knowledge on COVID-19 symptom and mode of spread among police officer in Benue State command, 2020

Knowledge Variables	Agreement (N = 305)
**Symptom of COVID-19**	
Diarrhea	29 (9.5)
Fever	283 (92.8)
Headache	257 (84.3)
Leg pain	26 (8.52)
Tiredness	210 (68.9)
Difficulty in breathing	300 (98.4)
Cough	301 (98.7)
**Mode of spread of COVID-19**	
Coughing and sneezing from COVID-19 suspected patient without covering the mouth	302 (99.0)
Disperse droplets into the air	261 (85.6)
Airborne	125 (41.0)
Touching or shaking infected person	298 (97.7)
Contact with eye/nose/ear/mouth	289 (94.8)

**Table 4:** risk perception of COVID-19 infection among police officer in MKD metropolis, 2020

Knowledge Variables	Agreement (N = 305) Frequency (%)
**Perception of risk of infection with COVID-19**	
Think COVID-19 exist	292 (95.7)
Think “as a police officer, I am at risk of COVID-19”	290 (95.1)
Think a healthy person could be infected with COVID-19	290 (95.1)
A good christian or muslim could be infected with COVID-19	260 (85.3)
Think COVID-19 preventable	282 (93.1)
**Perception on COVID-19 prevention**	
Wash hands regularly with soap, running water and hand sanitizer	297 (97.4)
Use of nose and face mask	278 (91.5))
Avoid overcrowded areas	298 (97.7)
Avoid touching your eye, nose and mouth	295 (96.7)
Avoid contact with contaminated surfaces, objects or items of personal use	294 (96.4)
Health education	288 (94.4)
Avoid hugging	293 (96.1)
Natural or herbal drugs	57 (18.7)
Use of charm	27 (8.83)
**Perception on behavioral risk for COVID-19 prevention**	
Willingness to change the way of life to prevent COVID-19 infection	299 (98.0)
Think suspected COVID-19 patient should share room with unexposed person	75 (24.6)
Will allow suspected COVID-19 patients share ones’ personal belongings	286 (93.8)
Think suspected COVID-19 people should be isolated	286 (93.8
Will continue friendship with a friend who recovered from COVID-19	213 (69.8)
Possibility of re-infection with COVID-19 after recovery from initial infection	239 (78.4)
Necessity of a Police Officer owning a personal protective equipment	288 (94.4)
Willingness to learn more about COVID-19	286 (93.8)

**Table 5:** adherence and outcome variables to COVID-19 prevention practices among police officer in MKD Metropolis, 2020

Adherence Variables	Agreement Frequency (%)
Wash your hands regularly with soap and water	284 (93.1)
Frequency of daily hand washing **(n = 284)**	
Once	7 (2.5)
Twice	32 (11.3)
More than twice	245 (86.3)
Own an alcohol-based hand sanitizer	175(57.4)
Use the alcohol based sanitizer during duty **(n = 175)**	167 (95.4)
Frequency of daily use of alcohol-based sanitizer **(n = 167)**	
Once	7 (4.2)
Twice	11 (6.6)
More than twice	149 (89.2)
Owns a face mask	107 (35.1)
Uses a face mask **(n = 107)**	94 (87.9)
Owns at least a pair of hand gloves	83 (27.2)
Uses a pair hand gloves during the arrest or touching of suspects **(n = 83)**	60 (72.9)
Disinfects riffle before and after usage	78(25.6)
Maintain social distancing during suspect arrest or interview	141 (46.2)
**Outcome Variables**	
Level of Knowledge on COVID-19	
Poor Knowledge	3 (1.0)
Good Knowledge	302 (99.0%)
Level of risk perception on COVID-19	
Negative risk perception	2 (0.7)
Positive risk perception	303 (99.3)
Level of adherence on COVID-19	
Poor adherence	172 (56.4)
Good adherence	133 (43.6)

Most participants demonstrate good knowledge on COVID-19 infection, 302 (99.0%) and positive risk perception of COVID-19, 303 (99.3%) but few demonstrated good adherences on COVID-19 prevention practices, 133 (43.6%) (Table 5). Participants´ academic qualification (X^2^ = 10.98, p = 0.001) and cadre (X^2^ = 112.5), p = 0.001) were found to be associated with good adherence to COVID-19 prevention practices. This was also true for owning an alcohol-based sanitizer, face masks and a pair of gloves (Table 6). Multivariate analysis revealed that owning an alcohol-based sanitizer (aOR = 0.008, 95% CI: 0.0016-0.0436), face masks aOR = 0.019, 95% CI: 0.0042-0.0852) and a pair of gloves aOR = 0.074, 95% CI: 0.0211-0.2611) by participants were associated with good adherence to COVID-19 advisory (Table 7).

**Table 6:** bivariate analysis of factors associated to poor adherence to COVID-19 advisory among police officers in Benue State, 2020

Variable	Level of adherence		Test statistic (x2)	P=value
	**Poor**	**Good**		
**Age group (years)**				
<=19	0	1	7.06	0.50
20 - 29	21	19		
30 – 39	77	47		
40 – 49	49	46		
50 – 59	15	20		
**Sex**				
Male	112	84	0.12	0.72
Female	60	49		
**Marital Status**			2.9	0.20
Married	143	107		
Single/Divorced/widow	24	25		
Co-habiting	5	1		
**Religious Status**				
Christianity	159	122	1.2	0.40
Islamic	11	11		
Others	2	0		
**Highest Academic Status**			10.98	0.01
		
Primary	2	0		
Secondary	103	57		
Tertiary	67	76		
**Family Size**			4.97	0.12
1-5	89	76		
6-10	79	52		
=>10	4	5		
**Cadre**			12.5	0.01
Commissioner	2	2		
Superintendent	19	35		
Inspectorate	61	42		
Rank & File	90	54		
**Own an Alcohol-based Sanitizer**			108.9	0.001
Yes	54	121		
No	118	12		
**Own a face mask**			131.2	0.001
Yes	13	94		
No	159	39		
**Own a pair of hand-gloves**			96.2	0.001
Yes	9	74		
No	163	59		

**Table 7:** multivariate analysis of factors associated to poor adherence to COVID-19 advisory among police officers in Benue State, 2020

Variables	Odds Ratio	95% Conf. Interval	P-value
Educational Status	0. 682	0.3024 -1.5379	0.356
Cadre	0.880	0.5293-1.4639	0.623
Owns an alcohol- based Sanitizer (yes/no)	0.008	0.0016-0.0436	0.001
Own a Face Mask (yes/no)	0.019	0.0042-0.0852	0.001
Own a Hand glove (yes/no)	0.074	0.0211-0.2611	0.001

**Figure 1:**
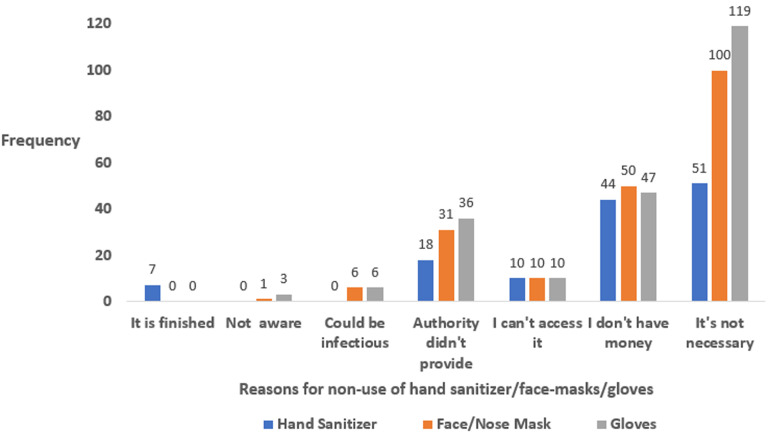
respondents’ reasons for not having hand sanitizer, face/nose mask and gloves

**Figure 2:**
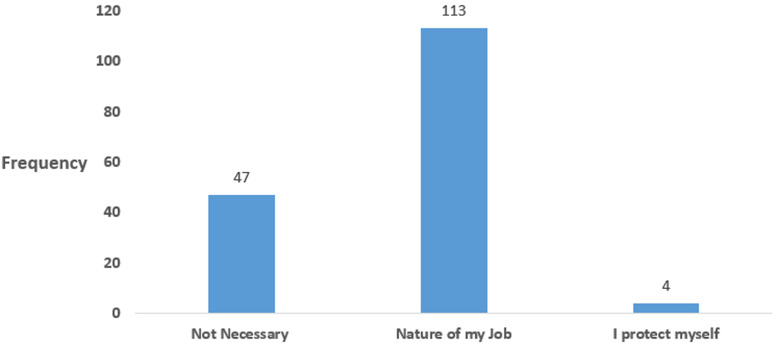
respondents’ reasons for not observing social distancing

Most participants´ reason for not having a hand sanitizer, face mask and a pair of gloves include not seeing it as necessary, lack of money and not being provided by the authority (Figure 1). The nature of job was the most common reason mention by participants for not observing social distancing while some do not consider it necessary (Figure 2).

## Discussion

Corona virus pandemic continues to threaten lives and greatly impact the global economy negatively. To contain this pandemic, accurate knowledge, risk perception and adherence to public health advisories is crucial. These variables were assessed among police officers in Benue State. Most of the police officers that participated in the study were within the age bracket of 35-39 years old, while the least age group comprised individuals less than twenty years old. Approximately two-thirds of the respondents were male. This may be related to the fact that more males work in the force compared to women. About half of the officers had attained a minimum of secondary education, while a small number had no formal education. Global reports have shown that level of education is a strong determinant of health behaviours and preventive service use. Furthermore, studies have shown that for education to be beneficial, at least secondary education should be attained.

Risk communication through reliable channels is essential during epidemics to dispel myths and rumours as well as provide accurate information on disease notification and treatment. In this study, nearly all (98.7%) of the respondents heard about COVID-19 from various media channels. Of these, television/radio accounted for the most common (76.4%) source of information. Similarly, in a study conducted in Nigeria by Olapegba *et al*. [22], TV/radio was the most common source (81.5%) of information [22]. This is contrary to the report by Kyaw *et al*. where social media accounted for the most common source of information (64.6%) [23]. This difference may be due to the ease of access of smart phones among the general population, unlike the force who use their smart phone when necessary during work hours [24].

In addition, a study in an adult US population showed that 80% of the participants mentioned healthcare professional as their primary source of COVID-19 information [25]. This difference may be due to variations in organization of the health system amongst these countries. It is pertinent to note that all age groups can be affected with COVID-19, although the diagnosis in children is challenging [10, 26] The major symptoms of COVID-19 such as fever and cough are typical presentations in children with common viral illness [9, 26]. In this study, about two-thirds of the respondents were aware that the disease affects all age groups. For effective control, it is important for public health messages to emphasize the potential for children to be affected and also serve as vehicles for disease propagation [9, 11, 16].

Knowledge of aetiology, risk factors and preventive measures of a disease has been intrinsically linked to good adherence [19]. This study demonstrated good knowledge of the aetiology of COVID-19 among the participants. More than two-thirds (65.6%) of respondents mentioned that COVID-19 is a viral infection. This finding is contrary to a study done by Olapegbe *et al*. [22] where nearly half (46.94%) of respondents stated that COVID-19 is a biological weapon. Also, in the Kyaw *et al*. study [23] less than a third (29.3%) of participants mentioned that COVID-19 is a viral infection. This difference may be due to the variations in primary source of information. Also, in the Kyaw *et al*. study, social media was the most significant source of information and two-fifth of respondents concurred to sharing unverified information on social media [23, 24]. The implication of having accurate knowledge of disease aetiology is that, when people know the scientific basis of a disease, they are more likely to have better-health seeking behaviours.

Conflicting information exists on the availability of curative drugs for COVID-19. Several drugs such as hydroxychloroquine, azithromycin and herbal remedies have been proposed as the mainstay of treatment [20]. In this study, about a third (29.5%) of the participants believe that COVID-19 has curative drugs while another third (32.13%) believed otherwise. A contrary report was obtained from Myanmar where about 70% of participants were aware that there was neither a vaccine nor definitive treatment for COVID-19 [23]. This difference may be due to the variations in focus of public health messages in the countries However, even though these claims on availability of a potent treatment were refuted by WHO, these contradictory information questions the credibility of the health system. This may accentuate the need for more stringent adherence to protocols such as that stipulated by the International Conference on Harmonization of Technical Requirements for Registration of Pharmaceuticals for Human Use (ICH) [27]. In addition, public health messages should periodically update the public on the status of drugs and vaccine undergoing clinical trial.

Several reports have stated that a significant proportion of COVID-19 cases may be asymptomatic [15, 28]. In a study conducted among passengers and crew on board an isolated cruise ship, the asymptomatic cases comprised four-fifth of the total population [28]. Asymptomatic transmission of SARS-CoV-2 has been described as the Achilles´ heel of COVID-19 pandemic control efforts [15]. In this study, three-quarters (75.08%) of the officers were aware that a significant proportion of COVID-19 infected persons may be asymptomatic. Similarly, 78% of respondents in a study conducted in Myanmar responded that a person can suffer from COVID-19 without signs and symptoms. The implication of this is to ensure people practice social distancing and hand hygiene to everyone they come in contact irrespective of their apparently healthy looks. Correctly identifying symptoms of the disease will enhance early intervention and improve contact tracing. Nearly all the police officers accurately mentioned respiratory symptoms like cough, difficulty in breathing and fever as the most common symptoms. This is in keeping with reports from WHO and CDC [6, 8, 11] where majority of people reported these symptoms. However, these symptoms are like features seen in other respiratory illnesses and endemic diseases such as malaria [6, 8, 10]. This implies that public health messages should emphasize the need for physicians and the public to have a high index of suspicion concerning any atypical or unexplained symptoms at this time.

Droplet infection and direct contact with mucous membrane of an infectious person or spread through fomites has been reported, as the most common routes of transmission of COVID-19 [6, 8, 9]. This is in line with the response of the participants in this study where nearly everyone knew that COVID-19 can be spread through droplets of an infected person. In addition, almost all the participants responded that direct contact through shaking hands and touching the mucus membranes with infected hands can spread COVID-19. Annals of pandemic control suggest that public adherence to public health advisories is directly linked to personal and community perceptions of risk factors [18, 19]. Nearly all the police officers believed in the existence of COVID-19, knew they can be at risk, and that the disease is preventable. In addition, an equal proportion were aware that healthy persons can be infected. This has positive implications on adherence to public health advisories.

Similarly, more than 90% of the officers believe that regular washing of hands with soap under running water, avoiding over-crowded areas and proper use of face mask can prevent COVID-19. This finding is similar to that of a study by Olapegba *et al*. [22] where 94% of respondents correctly reported handwashing and social distancing as preventive measures. These studies demonstrate good knowledge of preventive measures of COVID-19 among participants. Although knowledge does not necessarily translate to practice, the health belief model suggests that obtaining accurate information is the first step in behaviour change [19]. Although 93.1% of the respondents wash their hands with soap and water, only about half (57.4%) had alcohol-based hand sanitizer, suggesting that the general level of adherence to COVID-19 prevention advisory was poor. On the contrary, a study by Kyaw *et al*. [23] reported frequent handwashing with soap and water and alcohol-based hand sanitizer in only 54.8% of participants. This suggests a gap in hand hygiene practice because running water may not be easily accessible in some places. In these situations, hand sanitizer is meant to serve as an adjunct to running water. Thus, police officers had good knowledge of COVID-19 risk factors and transmission dynamics as well as good risk perception. However, adherence to public health advisories was poor. Educating the police officers on the need to purchase and use their personal hand sanitizer may be beneficial in reducing individual and community transmission of SARS-CoV-2 virus.

Disease surveillance and notification is an integral part of epidemic control [11]. More than three-quarters of respondent were knowledgeable about COVID-19 reporting process and had relevant reporting lines at their disposal. Prompt reporting of disease is crucial for early intervention and limitation of disease transmission. Police officers had good knowledge of transmission dynamics, good risk perception and adhered sufficiently to public health advisories. However, use of sanitizer was poor among them, suggesting the need for more training using virtual tools. Improving adherence behaviour among police officers is crucial in limiting the spread of the virus. If their risk perception and adherence is insufficient, they may serve as vehicles for propagating the spread of the virus in the course of their duties. Therefore, it is important to reinforce strategies to improve adherence such as periodic training of police officers and ensuring provision and use of adequate PPE and personal sanitizers. This may contribute in some measure to flattening the epidemic curve.

This study is the first to assess knowledge, risk perception and adherence to public health advisories among police officers, who are not just front-line workers in this pandemic response, but agents of change. However, even though police officers constitute a microcosm of the larger society, they represent a distinct occupational group; therefore, external validity in this study may be compromised. Also, we relied on self-reporting as against directly observing adherence behaviour. Consequently, the reported adherence may be exaggerated by the police officers.

## Conclusion

Adequate knowledge, risk perception and adherence to COVID-19 prevention advisories is important and key to containing and flattening the curve of COVID-19 infection. Our study is the first of its kind among police officers in Nigeria. The study revealed that the officers had considerable good knowledge and risk perception of COVID-19 infection, while their adherence to public health prevention advisories was poor. This is largely due to unavailability of PPEs such as personal hand-sanitizers, facemasks. The Police is a crucial and an essential workforce of every society, we recommend that basic PPEs to be provided for them so that the officers will not serve as vehicles for the transmission of COVID-19 infection among their families and the society they serve. The research outcome has been shared with the police authority in Benue State for implementation.

### What is known about this topic

- There is high COVID-19 awareness among the general population;
- Non-pharmaceutical intervention is effective in preventing COVID-19 infection.

### What this study adds

- The study added that there is good knowledge of COVID-19 among police officers and a high positive risk perception among police officers;
- The study added that there is poor adherence to COVID-19 adherence among police officers;
- The study indicates that highest academic status, cadre, ownership of alcohol-based sanitizer, face mask and hand gloves were associated to poor adherence to COVID-19 advisory.
